# Targeted therapy in chronic diseases using nanomaterial-based drug delivery vehicles

**DOI:** 10.1038/s41392-019-0068-3

**Published:** 2019-08-30

**Authors:** Akhand Pratap Singh, Arpan Biswas, Aparna Shukla, Pralay Maiti

**Affiliations:** School of Materials Science and Technology, Indian Institute of Technology (BHU), Varanasi, 221005 India

**Keywords:** Cardiovascular diseases, Diseases

## Abstract

The application of nanomedicines is increasing rapidly with the promise of targeted and efficient drug delivery. Nanomedicines address the shortcomings of conventional therapy, as evidenced by several preclinical and clinical investigations indicating site-specific drug delivery, reduced side effects, and better treatment outcome. The development of suitable and biocompatible drug delivery vehicles is a prerequisite that has been successfully achieved by using simple and functionalized liposomes, nanoparticles, hydrogels, micelles, dendrimers, and mesoporous particles. A variety of drug delivery vehicles have been established for the targeted and controlled delivery of therapeutic agents in a wide range of chronic diseases, such as diabetes, cancer, atherosclerosis, myocardial ischemia, asthma, pulmonary tuberculosis, Parkinson’s disease, and Alzheimer’s disease. After successful outcomes in preclinical and clinical trials, many of these drugs have been marketed for human use, such as Abraxane®, Caelyx®, Mepact®, Myocet®, Emend®, and Rapamune®. Apart from drugs/compounds, novel therapeutic agents, such as peptides, nucleic acids (DNA and RNA), and genes have also shown potential to be used as nanomedicines for the treatment of several chronic ailments. However, a large number of extensive clinical trials are still needed to ensure the short-term and long-term effects of nanomedicines in humans. This review discusses the advantages of various drug delivery vehicles for better understanding of their utility in terms of current medical needs. Furthermore, the application of a wide range of nanomedicines is also described in the context of major chronic diseases.

## Introduction

Nanomedicine is an emerging field that employs nanosized materials for applications in disease diagnosis and therapeutics. For example, nanotechnology-based methods and materials have been developed for the diagnosis and treatment of cancer.^1,2^ The combined application of nanoscience and pharmaceutical science is very promising and has grown rapidly in recent times. A wide variety of organic, inorganic, polymeric and metallic nanostructures, including dendrimers, micelles, solid lipid nanoparticles (SLNs), carbon nanotubes, and liposomes, are frequently used as targeted and controlled drug delivery vehicles.^3–5^ In particular, low-soluble drugs with poor absorption ability are encapsulated with nanomaterials for controlled and sustained drug release. However, the efficacy of these drug delivery vehicles depends on their size, shape, hydrophobicity, surface parameters, and several other chemical and biophysical features.^6,7^ Ideally, nanometer-sized materials with high biocompatibility^8^ and biodegradability^9^ are considered excellent drug delivery systems for biomedical applications.^10^ The overall surface area of the particles increases to several orders of magnitude higher when the particle size is on the order of nanometers, as opposed to the usual micron-sized particulate material used in conventional diagnostic and therapeutics. It is the surface area that is mainly interactive with its surroundings, and therefore, materials with nanometer dimensions have high potential for interaction with systems. Briefly, nanotechnology offers several advantages in treating chronic human diseases through sustained and targeted drug delivery. However, toxicity issues warrant extensive research to ensure high safety for the implementation of these medicines in clinical settings.^11^ Considering the promise of nanotechnology in the therapy of human diseases, this review aims to cover various nanomaterial-based drug delivery systems, their applications in the diagnosis and therapeutics of chronic diseases, and other associated challenges.

## Drug delivery systems

Drugs and other therapeutic agents are administered to treat specific diseases and disorders with the goal of achieving desired pharmacological effects with minimum side effects. The application of a controlled drug delivery system is a central strategy to enhance the therapeutic efficacy and safety of therapeutic molecules.^12,13^ The primary rationale of using a suitable drug delivery system is its ability to ensure a higher and longer duration of drug bioavailability and thereby enhanced therapeutic efficacy.^14,15^ Various materials with different structural forms are conjugated with drugs to prepare nano drug delivery systems. Considering recent approaches, most commonly used drug delivery vehicles include nanoparticles (e.g., polymeric, ceramic, and metallic),^16^ liposomes,^17^ micelles,^18^ and dendrimers,^19^ etc. A substantial number of preclinical and clinical studies suggest their suitability for the treatment of various diseases.^20–22^ The number of materials for use in drug delivery applications is rapidly increasing, and such materials have shown great diagnostic and therapeutic potential.^23,24^ A detailed description of promising drug delivery vehicles is given below.

### Hydrogel

Hydrogels or hydrophilic gels are physically or chemically cross-linked polymeric networks that exhibit the ability to swell in the presence of water or organic solvents. Hydrophilic functional groups attached to the polymeric backbone are credited with its ability to absorb water, and the resistance to dissolution is due to cross-linked polymeric networks.^25^ Hydrogels can be synthesized in a number of ways, including one-step or multistep procedures. A one-step procedure involves the polymerization and parallel cross-linking of multifunctional monomers, while a multiple-step procedure employs the synthesis of polymer molecules with reactive groups and their subsequent cross-linking with suitable agents.^26^ In the case of physical cross-linking, polymers are swelled in water to produce a network structure, mainly through extensive hydrogen bonding. Based on the polymer source, hydrogels may be homopolymer, copolymer or multiblock in nature.^27^ Usually, hydrogels are prepared through the copolymerization reaction of hydrophilic monomers and multifunctional cross-linkers. There are several methods for hydrogel preparation, including bulk polymerization,^28^ solution copolymerization,^25^ suspension polymerization,^29^ and irradiation polymerization.^30^ In bulk polymerization, hydrogels are prepared by using any suitable monomer in high concentration with a small amount of cross-linking agent. Furthermore, radiation or chemical catalysts may be used for the initiation of the polymerization reaction. Solution copolymerization involves the mixing of ionic or neutral monomers with multifunctional cross-linking agents. The solvent used during the polymerization process acts as a heat sink, which is a major advantage of this method over bulk polymerization. Furthermore, suspension polymerization has the added advantage of obtaining the products in the form of powder or microspheres (MS). This method uses water in oil instead of the more common oil-in-water method and is also referred to as inverse suspension polymerization.^25^ Hydrogels are biocompatible, nontoxic, and biodegradable with high absorption capacity, which ensures a wide range of biomedical applications in drug delivery, tissue engineering, and wound dressing/healing. Furthermore, stimuli-responsive materials such as pH- and temperature-sensitive smart hydrogels offer site-specific targeted drug delivery applications.^31^

### Micelle

Micelles are formed due to the dispersion of amphiphilic molecules, consisting of hydrophobic and hydrophilic components, in solution. The factors that govern the formation of micelles include the concentration of amphiphiles, hydrophobic/hydrophilic domain size in the amphiphilic molecule, temperature, and solvent.^32^ Micelles are formed through self-assembly, and the process starts only when a certain minimum concentration is achieved, often known as the critical micellar concentration. Furthermore, the temperature above which the amphiphilic molecules exist as aggregates is known as the critical micellization temperature, and micelles may collapse below this temperature.^33^ Polymeric micelles are of much interest due to their relatively high stability, minimal cytotoxicity and suitability for controlled and sustained drug delivery. Suitable micelles can be obtained by adjusting the monomer ratio in block copolymers. Most hydrophobic drugs may easily be incorporated into the cores of micelles. The nanoscopic core size and hydrophilic shell minimize the clearance of micelles from the body and eventually prolong the bioavailability of the drug. Polymeric micelles favor targeted therapy and sustained drug delivery due to the high drug loading capacity of the inner core. The hydrophobic micellar core is suitable for the incorporation of hydrophobic drugs. Furthermore, end functionalization of block copolymers with peptides, sugars and other moieties may be used for receptor-mediated targeted drug delivery. Polymeric micelles can be formed via several mechanisms, including hydrophobic interactions, electrostatic interactions, and metal complexation.^34^ Polymeric micelles are mostly prepared by the dissolution of the block copolymer in a selective/nonselective solvent, while drug-loaded micelles are mainly prepared by direct dissolution, solvent evaporation, and dialysis. For targeted therapy, several approaches use micelles, such as the enhanced permeability and retention effect, stimuli sensitivity, and ligand-based micelles, etc. In a stimuli-sensitive approach, a drug is released under internal stimuli (e.g., pH and enzyme) or external stimuli (e.g., light and temperature). For example, a pH-sensitive micellar system may be used in drug delivery to tumors, as reports confirm a mildly acidic pH (~6.8) in the tumor microenvironment.^35^ Similarly, thermosensitive and ligand-based micellar systems are also used for targeted drug delivery. Liu et al. showed the hyperthermia-mediated tumor targeting of docetaxel. Due to their nanosize, polymeric micelles may accumulate passively at the interstitial spaces of cancer or tumor tissue. The hyperpermeability of tumors leads to enhanced retention of micelles and thereby the accumulation of the incorporated drug within the tumor.^36^ Vetvicka et al. prepared a doxorubicin-conjugated poly(ethylene oxide)-b-poly(allyl glycidyl ether) micellar system that showed prolonged circulation and efficient release of doxorubicin at the tumor site.^37^ Similarly, Watanabe et al. developed a camptothecin-loaded micellar formulation using poly(ethylene glycol)-poly(aspartate ester) block copolymers for enhanced permeability and retention-mediated drug delivery.^38^

### Dendrimer

Dendrimers are radially symmetrical, globular and nanodimensionally compact structures with tree-like branches or arms. These hyperbranched macromolecules with a high number of functional groups have become an ideal delivery vehicle for a wide range of diagnostic and therapeutic applications. The dendrimer structure consists of a central core composed of an atom or group of atoms from which branches of other atoms grow through a series of chemical reactions.^39^ Primarily, dendrimers are synthesized either by divergent or convergent methods. In a divergent approach, the synthesis cascade starts from the core and extends towards the periphery in a stepwise manner.^40^ As such, a first-generation dendrimer is synthesized when a multifunctional core molecule reacts with monomer molecules composed of one reactive and two dormant groups. In the next step, more monomers become involved in the synthesis of new peripheral arrangements via a cascade of reactions. In the convergent method, synthesis starts from the periphery, and the start point becomes the outermost layer of the final dendrimer at the end.^40^ It is worth mentioning that several modifications to the peripheral groups of dendrimers are available, which enable them to be ready for a wide range of biomedical applications. The most promising application of dendrimers lies in their ability to perform controlled and targeted drug delivery.^41^ Drugs conjugated to such delivery vehicles are characterized by higher stability, increased half-life and bioavailability. Furthermore, sustained drug release through the drug-dendrimer conjugate reduces systemic toxicity and maintains selective accumulation in tumor tissue. Different methods of encapsulation, complexation, and conjugation are used to tag genetic materials, drug molecules and dyes for diagnostic and therapeutic applications. Dendrimer-based metal chelates are used as imaging contrast agents during MRI.^42^ Dendrimers have been proven to be an efficient transdermal delivery vehicle for drugs with low solubility and highly hydrophobic moieties.^43^ Dendrimers have also been applied in the photodynamic therapy of tumors, where it facilitates the release of highly reactive singlet oxygen, which capable of inducing apoptosis and necrosis in tumor cells upon excitation.^44^

### Nanoparticle

The role of nanoparticles as therapeutic carriers has been extensively investigated, as evidence by a substantial number of clinical and preclinical investigations. Nanoparticles are considered to be among the cohort of promising drug delivery vehicles. Polymeric nanoparticles are synthesized via the self-assembly of amphiphilic copolymer chains in aqueous media.^45^ Several hydrophilic and hydrophobic blocks of different structures, lengths and charges have been used to prepare polymeric nanoparticles of various shapes, sizes, and stabilities. As a result, these nanoparticles are able to encapsulate both hydrophilic and hydrophobic drugs as well as to bind macromolecules, such as proteins, antibodies or nucleic acids.^46^ Genexol-PM has been prepared by the encapsulation of paclitaxel in polymeric nanoparticles formed by the block copolymer of monomethoxy poly(ethylene glycol) and poly(d,l-lactide).^47^ Nanoparticles may also be designed to respond to external stimuli, including light, temperature, enzyme, pH, and other biological and chemical agents.^48^ Among all these stimuli, pH responsiveness is the most frequently used stimulus. pH helps to differentiate normal tissue (pH, 7.4) from tumor tissue (pH, 5.7–7.0). This pH responsiveness has been utilized in various cancer/tumor treatments with the advantage of drug release specifically at tumor sites.^49^ Apart from polymeric nanoparticles, inorganic nanoparticles have also been extensively investigated for their biomedical applications.^45^ Inorganic nanoparticles are prepared using several methods, such as the crystallization of inorganic salts, thermal decomposition and other established synthetic processes.^50^ Because of the simplicity of the reaction process, the thermal decomposition method is usually used for the mass production of stable inorganic nanoparticles. In the thermal decomposition process, metal precursors are added to organic solvents in the presence of stabilizers.^51^ Various inorganic nanoparticles (e.g., silver, gold, platinum, zinc oxide, iron oxide, and cerium oxide) have been successfully prepared and employed in various preclinical and clinical studies. However, polymeric nanoparticles are preferred over inorganic nanoparticles due to their high biocompatibility, high biodegradability, and reduced systemic toxicity.^52^

### Liposome

Liposomes have also attracted much attention as drug delivery systems due to their biocompatibility, biodegradability, low toxicity, and site-specific delivery of both hydrophilic and hydrophobic drugs. Liposomes are considered promising drug delivery vehicles due to their small size, biocompatibility, and chemical nature. Liposomes are small, spherical amphipathic (both hydrophilic and hydrophobic) vesicles composed of phospholipids. Liposomes differ considerably in their properties depending upon lipid composition, size, surface charge and preparation methods.^53^ Liposomes can encapsulate both hydrophilic and hydrophobic drugs, prevent the rapid decomposition of the encapsulated drug, and eventually release the drug molecules at designated targets. Furthermore, the rigidity or fluidity and surface charge of liposomes are determined by the choice of bilayer components. The vesicle size and number of bilayers determine the amount of encapsulated drug, and on the basis of their size, bilayer liposomes are subdivided into two categories: (1) multilamellar vesicles and unilamellar vesicles. Unilamellar vesicles can further be classified as large unilamellar vesicles and small unilamellar vesicles. Unilamellar vesicles consist of a single phospholipid bilayer enclosing the aqueous solution, while multilamellar vesicles consist of concentric phospholipid spheres separated by water layers.^21^ As such, liposomes are prepared by dispersing the lipid in aqueous media. Furthermore, dispersion is performed through several methods, including mechanical (sonication and microemulsification) and solvent dispersion methods.^53^ Currently, stealth liposomes are preferred over conventional liposomes due to their stability in the bloodstream because they can escape the mononuclear phagocytic system, which engulfs and clears liposomes from circulation. Reports also suggest that coating liposomes with PEG reduces the uptake by macrophages and ultimately favors its prolonged presence in the bloodstream. Stealth liposomes have been used successfully in the delivery of doxorubicin for the treatment of solid tumors and are presently marketed as ‘Doxil’ or ‘Caelyx’.^54^

### 2.6. Mesoporous material

**Fig. 1:**
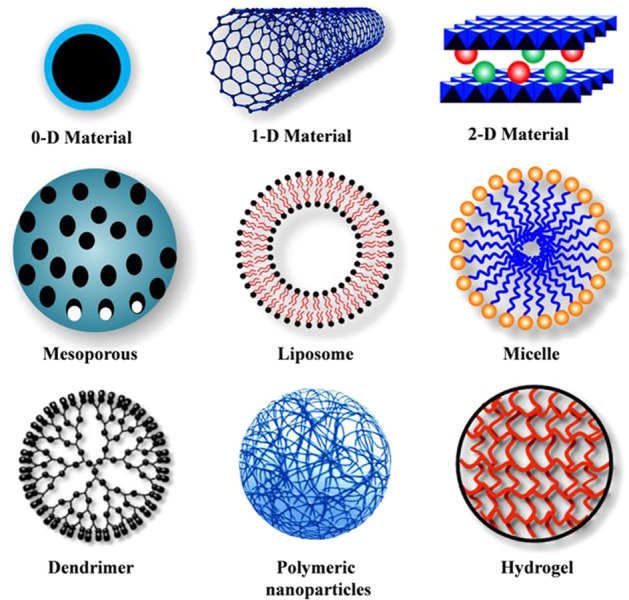
Different types of nanocarriers used as controlled delivery vehicles for therapy. Reproduced from ref. ^7^

Efficient drug loading and controlled release at the desired site can also be achieved by using mesoporous materials. Furthermore, pore size, surface functionalization, and surface area are the main factors influencing drug loading and release behavior.^55^ There are several methods for the synthesis of mesoporous materials, such as the sol–gel method, hydrothermal synthesis, microwave synthesis, and template synthesis.^56^ The synthesis of mesoporous silica is accomplished using sol–gel chemistry in the presence of surfactants. Furthermore, thermal crosslinking rigidifies the preceramic material, and thereafter, the surfactant is removed to expose the pore structure.^57^ In addition, various synthetic methods have been employed for the preparation of mesoporous ceramics. Mesoporous materials with different shapes, sizes and symmetries of mesoporous materials can be synthesized by varying the reaction conditions. Cubic and hexagonal phases could be obtained simply by exposing the materials to specific reaction conditions. Several simple and less time-consuming methods are being developed for the synthesis of mesoporous materials. Currently developed block copolymer synthesis in acidic conditions is more rapid (6–12 h) than conventional methods (1 week). Furthermore, advanced methods such as microwave heating reduced the synthesis time to less than an hour along with control over the structure of the mesoporous ceramics.^58^ Microwave synthesis is an efficient method to prepare ordered mesoporous materials, such as MCM-48, MCM-41, and SBA-15.^56^ Figure 1 represents the state of various drug delivery vehicles for disease control.

### Scaffold

The application of scaffolds in biomedicine and tissue engineering is of vital importance because scaffolds promote cell adhesion, proliferation, and differentiation for tissue growth. Scaffold provides a porous network for the optimum growth of cells, eventually leading to the formation of tissue. The approach of using scaffolds is of great importance because a biocompatible biomaterial matrix is needed for optimum cell growth.^59,60^ In tissue engineering, scaffolds are designed to offer structural support similar to that of naturally occurring extracellular matrix. Scaffolds are employed in the position of natural fibrous collagen during bone tissue engineering, which plays a vital role in bone regeneration by regulating cell adhesion, proliferation, and differentiation.^61^ Importantly, scaffolds offer key morphological and mechanical characteristics for clinical application, such as high porosity, interconnectivity, and mechanical strength, along with high biocompatibility.^62^ Currently, in comparison to traditional solid surface scaffolds, nanofibrous scaffolds have shown potential application in tissue regeneration compared to traditional solid surface scaffolds, enabling the precise replacement of damaging tissue. The scaffold of particular interest can be designed primarily by varying the solvent and polymer concentration, which provides a scaffold construct with specific size, shape, and porosity. However, porogens are also used to control the pore size, shape, and interconnectivity.^63^ Considering biomedical applications, a substantial number of conventional techniques have been developed to achieve scaffold-based drug delivery. The conventional techniques for scaffold fabrication, such as particulate leaching or solvent casting, are intended to form the desired scaffold shape and porosity but are mostly limited with respect to interconnectivity or internal fiber design.^64^ An appropriate solvent in combination with homogenous salt particles is used to dissolve the polymer solution for the solvent-casting method. Furthermore, the solvent evaporates, leaving a salt particle-containing matrix that forms a highly porous structure after the water-mediated leaching out of salt particles. The solvent casting method is appropriate only for thin films or membranes because the soluble particles can be leached out through a thin polymer matrix. Scaffold preparation using this method results in high porosity (50–90%), which is considered suitable for the development and growth of the cell.^65^ Freeze-drying or lyophilization is another method that involves the dissolution of polymers in an appropriate solvent. The prepared solution is cooled under the freezing point, and sublimation/evaporation of solid solvent leaves a solid scaffold with a high number of interconnected pores. The benefit of the freeze-drying technique is its potential to maintain the activity of integrated biological factors that might otherwise decrease at high temperatures.^66^ Additionally, the pore size can be tuned by altering the freezing temperature. Although this technique is widely utilized for scaffold fabrication, the use of cytotoxic solvents and the generation of small and irregular pores limits its application.^67^ The limitations of high temperature and cytotoxic organic solvents may be alleviated by using the gas foaming technique. This technique uses relatively inert gas foaming agents, such as carbon dioxide or nitrogen, to produce sponge-like structures with high porosity (up to 85%) and a wide range of pore sizes (30–700 μm).^67,68^ Furthermore, an electrospinning approach is utilized for making nanofibrous scaffolds from a solution by applying high potential. Electrospinning is a quick method to make fibers with high tensile strength that has several applications in biomedicine and tissue engineering.^69^ A few other methods are also employed for the preparation of various forms of scaffolds, such as rapid prototyping, stereolithography, fused deposition modeling, selective laser sintering, and bioprinting.^67^

## Application of various nanocarriers for diagnosis and treatment

The nanocarriers discussed above are being used for diagnosis and disease control. Various diseases that can be detected and subsequently treated using nanomedicines are discussed below.

### Nanomedicines in diabetes management

In the last few decades, diabetes mellitus (DM) has become a chronic metabolic disorder that has impacted the lifestyle of billons of peoples throughout the world. DM ranks among the top five reasons for death in most developed and developing countries.^70^ According to the International Diabetes Federation, the number of people affected with diabetes worldwide is 382 million, which has been estimated to reach 592 million in 2035.^71^ In general, DM is a chronic hyperglycemic situation that involves multiple etiologies, such as inappropriate protein, fat, and carbohydrate metabolism. Broadly, DM can be divided into two subcategories, i.e., type 1 DM (T1DM) and type 2 DM (T2DM). The absolute deficiency of insulin is the prime reason for T1DM, while T2DM is caused by a variable degree of insulin resistance, impaired insulin secretion, and greater glucose production. T1DM is further subdivided into types 1A and 1B. The autoimmune destruction of β cells belongs to 1A, while idiopathic insulin deficiency belongs to type 1B. The hyperglycemic condition in DM may result in chronic vascular effects such as nephropathy, neuropathy, stroke, cardiovascular disease, kidney damage, and fatty liver.^72,73^ For the management of diabetes, a monitoring programme is necessary to help to control the conditions and consequence complications through the adoption of a proper diet, regular physical exercise, and adherence to medication, if needed.^74,75^ In conventional therapy, oral hyperglycemic agents, and parenteral preparations of insulin and glucagon-like-peptide-1 receptor are generally used.^76^ Insulin is produced by β cells of the pancreas and enters the blood through exocytosis. It is a polypeptide hormone consisting of 51 amino acids in two chains, one chain of 21 and the other of 30 amino acids, connected through a disulfide linkage. The purpose of insulin therapy is to provide insulin replacement as close as possible to the site of action, and nanosized particles such as liposomes, micelles, and transmucosal patches may ensure site-specific drug delivery.

Liposomes are bilayers of one or more phospholipids and are produced from nontoxic phospholipids and cholesterol.^53^ They are generally biocompatible, biodegradable, nontoxic, and able to entrap lipophilic and hydrophilic drugs for site-specific drug delivery.^77,78^ Zhang et al. modified the liposome to improve site-specific insulin delivery. The liposome with a lipid:cholesterol ratio of 3:1 shows maximum insulin trapping efficiency along with good membrane fluidity and minimum insulin leakage from internal aqueous components.^79^ In an alternative approach, chitosan-coated liposomes facilitate insulin delivery, and the hypoglycemic efficacy increases with increasing chitosan molecular weight.^80^ Furthermore, liposomes tagged with folic acid improve the targeted delivery of insulin and anionic poly(acrylic acid) and cationic poly(allyl amine) hydrochloride coatings to stabilize the liposomes. A sustained hypoglycemic condition was observed for 18 h after an advanced dose.^81^ Niosomes are like synthetic microscopic vesicles with nanoscale dimensions. They consist of nonionic surfactants impregnated with cholesterol as an excipient.^82^ Niosomes are capable of delivering various drug molecules in a sustained manner and can accommodate a variety of drugs due to the presence of hydrophilic, amphiphilic and lipophilic moieties in their structure.^83^ Niosomes are usually prepared through lipid-phase evaporation techniques. Span 40 and Span 60 vesicles have shown their effectiveness in reducing blood glucose levels; however, Span-40-based vesicles have been found to be more effective. In another study, metformin hydrochloride was loaded in a Span-40-based niosome through a reverse-phase evaporation technique. Pharmacokinetic testing confirmed the better efficacy of metformin-loaded niosomes over pure metformin in reducing blood glucose levels.^84^

**Fig. 2:**
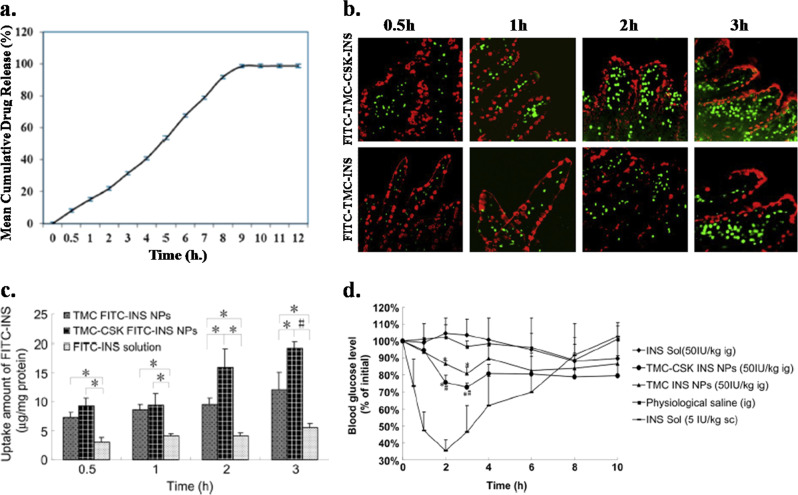
**a** In vitro drug release profile of insulin from PVA-alginate composite nanofiber patch. **b** Fluorescence images of the localization of FITC-TMC-CSK-INS and FITC-TMC-INS nanoparticles in the villi of the ileum after the indicated time, where green fluorescence represents FITC-loaded nanoparticles, while red fluorescence represents mucus droplets of goblet cells. **c** Quantification of the uptake of insulin-loaded nanoparticles and pure insulin by the HT29-MTX cells after different incubation times. (Mean ± SD, *n* = 3–5) *P < 0.05, ^#^*P* < 0.001. **d** Glucose levels in the blood of diabetic rats at different time intervals after the oral administration of different nanoparticles and insulin. The insulin concentration was maintained at 50 IU/kg. (Mean ± SD, *n* ~4). Significant differences from insulin solution (*) and from TMC-INS nanoparticles (#): *P* < 0.05. Reproduced with permission from refs ^85,86^

A biodegradable nanofiber-based transmucosal patch has been developed through the electrospinning of poly(vinyl alcohol)–sodium alginate composite, and the patch has been impregnated with the antidiabetic drug insulin through active loading for the management of diabetes.^85^ The in vitro drug release study confirmed a sustained and controlled release pattern of the insulin from the nanofiber (Fig. 2a) and ~100% drug release from the patch occurred within 12 h. The release pattern follows first-order kinetics with an initial burst release of the drug. The in vivo study confirmed the effectiveness of the drug-loaded nanofibers, as a significant lowering of the blood glucose level was observed in diabetic male Wistar rats. Furthermore, the patch showed better efficiency in controlling diabetes than did the commercial formulation. The chitosan nanoparticles were modified with trimethyl chloride (TMC) and then attached to the targeting peptide CSKSSDYQC (CSK) for better uptake of the nanoparticles by the villi. These modified chitosan nanoparticles are developed for goblet cell-targeted insulin (INS) delivery.^86^ The size of the nanoparticle was increased from 182 to 342 nm after the nanoparticles were modified with TMC, CSK, and INS, which decreased the zeta potential from 10 to 3 mV. Qualitative observation of the absorption of the nanoparticles in the villi of the ileum by CLSM before and after tagging with the targeting peptide (CSK) is shown in Fig. 2b. The nanoparticles were modified with FITC fluorescent agent to obtain fluorescence images. Increased absorption was observed for both types of nanoparticles with time, and the maximum absorption was observed at 3 h. Furthermore, greater absorption of the nanoparticles modified with CSK peptide than of unmodified nanoparticles, which signifies that the CSK peptide promotes the absorption of the nanoparticles. Therefore, a targeted delivery of insulin is possible after attaching the nanoparticles to CSK. The quantification of cellular uptake of the insulin-loaded nanoparticles was performed using HT29-MTX cells, as shown in Fig. 2c. The uptake of pure insulin- and insulin-loaded modified nanoparticles increased with time, and the highest uptake was observed for nanoparticles modified with TMC and CSK, followed by nanoparticles modified with TMC alone. The least uptake was observed for pure insulin. The pharmacological study was performed on diabetic rats with TMC-CSK-INS, TMC-INS, physiological saline, and insulin, as shown in Fig. 2d. Both types of nanoparticles, TMC-CSK-INS and TMC-INS, showed better hypoglycemic effects than saline and insulin after 2 and 3 h of administration. TMC-CSK-INS nanoparticles showed the best hypoglycemic effect with a maximum depression of blood glucose by ~28% at 3 h, while TMC-INS nanoparticles showed ~20% depression of the glucose level at 3 h. Hence, the presence of CSK peptide increases the bioavailability of the insulin-loaded nanoparticles at the targeted site, which results in effective depression of the blood glucose level. Chalasani et al. reported that vitamin B12-coupled modified dextran nanoparticles of varied cross-linking, significantly improved the oral delivery of insulin.^87^ In diabetic rats, these vitamin B12-NPs conjugate effectively and reduce the blood glucose level (~70–75%) for an extended period of time (54 h) with biphasic behavior. Similar efficacy is also observed in diabetic mice, in which these NPs reduce the 60% blood glucose level at 20 h.

Cell-based therapy is considered one of the alternatives to insulin-based therapy for the treatment of diabetes. It includes β-cell regeneration, transplantation of insulin-producing cells to reestablish the insulin production system, and reprogramming the native cells to secrete insulin.^88^ Islet transplantation for type 1 diabetes is one type of exogenous cell therapy that is actually an approach to reestablish normoglycemic conditions in patients.^89^ However, it has very limited applications because of the tendency toward rejection of the transplanted cells by the host.^90^ The development of a bioartificial pancreas may be an alternative to immunosuppressive therapies, and advancements in nanotechnology may be very helpful in this context.^91^ Another alternative strategy is gene therapy, which either induces or silences immune response-specific genes to avoid the immunogenic problems associated with cell therapies.^92^ Different nanoparticles have been developed for the targeted delivery and protection of nucleic acids.^93^ DNA encoding interleukin (IL)-10 and IL-4 encapsulated in polymeric nanoparticles has been delivered into white blood cells to inhibit the T cell response against residual native islet cells in a prediabetic animal model. It was found to be very helpful in diabetic management because it inhibited diabetes development in 75% of the animals.^94^ However, the treatment of DM using nanoparticle-based vehicles is quite effective compared to conventional treatment because of sustained drug release from the vehicle.

### Neurological disease control using nanomaterials

Globally, the most common neurodegenerative diseases include Alzheimer’s disease (AD) and Parkinson’s disease (PD). The common clinical hallmark of AD is the extracellular deposition of amyloid beta (Aβ) peptide along with phosphorylated tau protein, which leads to irreversible neuronal loss and thereby to loss of memory and decision-making power.^95,96^ Furthermore, the degeneration of dopaminergic neurons of the brain followed by the inhibition of dopamine leads to PD. Bradykinesia, postural instability, rigidity, and resting tremor are the most common clinical symptoms that characterize the disease. The currently available pharmacological treatment for AD primarily includes the acetylcholinesterase (AChE) inhibitors tacrine, donepezil, rivastigmine (RT), and galantamine.^97^ AChE inhibitors improve postsynaptic stimulation by maintaining the levels of acetylcholine.^98^ Furthermore, a high level of glutamate tends to overstimulate neurons and eventually damage them via induced excitotoxicity. Memantine (a glutamate receptor antagonist) is a promising drug molecule that inhibits excess glutamate activity, which is important for memory and learning.^99^ On the other hand, dopamimetic drugs such as levodopa and carbidopa are prescribed for PD patients.

#### Nano drug delivery system for Alzheimer’s disease

Serious efforts have been made by the scientific community to develop potent molecules and compounds that could mitigate the progression of neurodegenerative diseases. The inability or limited ability to cross the blood–brain barrier (BBB) is the major reason for the limited therapeutic effects of the currently available drug molecules. However, nanomedicines are emerging as a novel strategy to overcome the BBB and to provide the advantage of targeted and sustained release. Additionally, bioavailability for longer durations limits the frequency of dosing and minimizes possible side effects. In preclinical studies, RT-loaded poly(lactide-co-gycolide) (PLGA) and polysorbate 80 (PBCA-80)-coated poly(n-butylcyanoacrylate) nanoparticles have shown potential in brain targeting.^100^ These nanoformulations improved the memory faster than RT in solution, as confirmed by the Morris Water Maze test.^100^ After intravenous administration of RT-loaded PBCA-80 NP, the uptake of RT was reported to be 3.82-fold higher than that of the free drug.^101^ Furthermore, RT-loaded chitosan NPs also improved memory function with reduced toxicity in a mouse model.^102^ In another study, donepezil-encapsulated PLGA NPs showed higher accumulation than the free drug in the brain. Donepezil nanoformulation demonstrated a biphasic release pattern characterized by an initial burst release followed by a sustained release. Investigators have suggested that the Tween 80 coating on these NPs improves brain-targeted drug delivery by facilitating the opening of the BBB.^103^ Galantamine is also used for the treatment of AD, but the lower bioavailability of the free drug limits its effectiveness. Interestingly, SLNs loaded with galantamine hydrobromide demonstrated enhanced bioavailability (two times higher than that of the pure drug) and improved memory and cognition in animals.^104^ The maximum drug entrapment of these SLNs was ~83 %, with greater than 90% drug release in 24 h. Hence, SLNs can be a promising vehicle for safe and effective brain targeting in diseases such as Alzheimer’s disease. Several cell line studies have also confirmed the effectiveness of a wide range of nanoformulations. PEGylated nanolipsomes and SLNs loaded with galantamine^105^ and memantine^106^ showed increased brain-targeted delivery with neuroprotective properties.^106^ Considering most recent therapeutic approaches in AD growth factors, delivery through nanoparticles is quite promising. In the APP/PS1 mouse model for AD, mice administered vascular endothelial growth factor-loaded PLGA nanospheres demonstrated excellent improvements in memory by the activation of neuronal progenitor cells in the hippocampal region.^107^

**Fig. 3:**
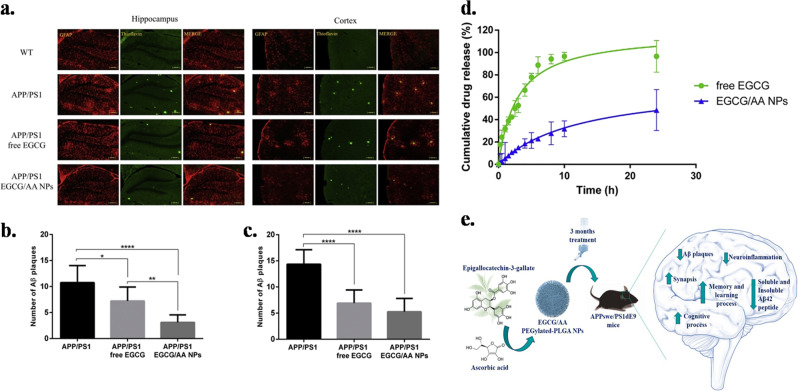
Therapeutic effects of EGCG/AA NPs in the APP/PS1 mouse model of AD. **a** GFAP and ThS immunostaining indicate the status of neuroinflammation and Aβ plaque deposition, respectively. Bar diagrams demonstrate the reduction in Aβ plaque deposition at the **b** hippocampus and **c** cortex area. **d** Percentage cumulative drug release showed sustained drug release of EGCG/AA NPs over free EGCG. **e** Schematic diagram of therapeutic effects of EGCG/AA NPs in an AD mouse model. Data are expressed as the mean ± SEM *P < 0.01, **P < 0.01, ***P < 0.001, ****P < 0.0001. Reproduced from ref. ^114^

Nerve growth factor is essential for the survival of neurons and may be utilized as a potential therapeutic strategy in neurodegenerative disorders. Nerve growth factor (NGF) does not cross the BBB efficiently, but a suitable drug delivery vehicle has been found to enhance its transport through the BBB. NGF adsorbed on PBCA-80-coated poly(butylcyanoacrylate) reversed scopolamine-induced amnesia and slowed the progression of neurodegeneration in a rat model.^108^ Furthermore, NGF loaded in transferrin- and cereport-functionalized liposomes enhanced the permeability across the BBB. Interestingly, the encapsulation of curcumin and NGF in combination led to a synergistic effect and showed a clinical application in mitigating Aβ-induced neurotoxicity.^109,110^ In another novel approach, antioxidant molecules, such as resveratrol, have also been delivered to the brain through nano drug delivery systems. An in vivo study suggested that resveratrol-encapsulated lipid core nanocapsules are highly effective against Aβ-induced neurotoxicity. The promising improvement in short-term and long-term memory was attributed to the higher bioavailability of resveratrol in the brain.^111^ In another study, curcumin-loaded lactoferrin-functionalized nanolipid carriers enhanced brain uptake by 2.78-fold and could recover hippocampal damage in the AD animal model.^112^ In cell culture, the nanostructured lipid carrier-mediated controlled delivery of resveratrol and curcumin showed synergistic effects against oxidative stress-induced neurodegeneration.^113^ In an interesting in vivo study, oral delivery of epigallocatechin-3-gallate (EGCG) NP formulation in an APP/PS1 AD mouse model reduced neuroinflammation and AB plaque (Fig. 3a–c). EGCG in combination with PEGylated PLGA NPs (EGCG/AA NPs) displayed increased stability and sustained release with high bioavailability compared to that of free EGCG. Furthermore, these data were supported by significantly improved spatial learning and memory (Fig. 3d, e).^114^

#### Nano drug delivery system for Parkinson’s disease

Dopamine (DA) and DA agonists are the most frequent treatment strategies in PD patients. However, limited permeability across the BBB restricts the therapeutic potential of these dopaminergic molecules. Furthermore, frequent doses are necessary to ensure high bioavailability, which eventually leads to systemic side effects such as nausea and dyskinesias.^115^ Nano drug delivery systems are quite promising to overcome these limitations, as confirmed by several studies. DA-loaded chitosan NPs facilitate transport across the BBB and minimize the cytotoxicity. A dose-dependent increase in dopamine level was observed after the administration of these NPs.^116^ Furthermore, intracranial implantation of scaffolds embedded with DA-loaded cellulose acetate phthalate NPs resulted in sustained drug delivery. The maximum DA entrapment efficiency was 63%, and the DA peak was reported to be highest at day 3 in both the cerebrospinal fluid and plasma of rats while maintaining an adequate level up to 30 days compared to inherent DA levels.^117^ Intrastriatal implantation of silica-DA nanoformulation also reversed the symptoms in hemiparkinsonian rats with no observable side effects.^118^

**Fig. 4:**
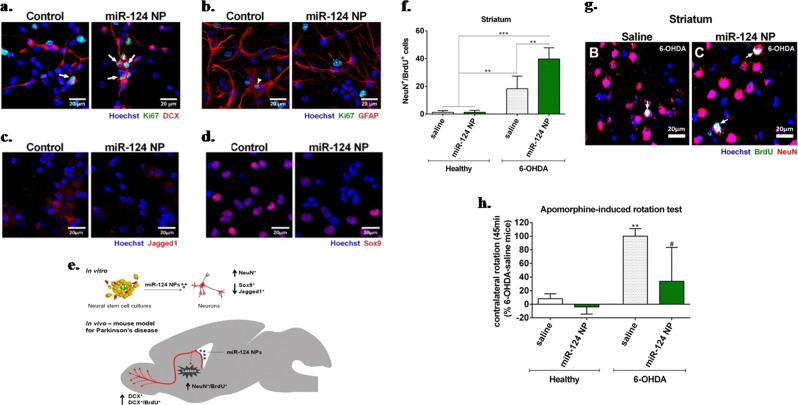
MiR-124 NPs promote neurogenesis in PD. **a** DCX/Ki67 and **b** GFAP/Ki67 and in miR-124 NP-treated cultures. **c** Jagged1. **d** Sox9. **e** Schematic representation of miR-124 NP effects in in vitro and in vivo study. **f** Therapeutic effect of miR-124 NPs on the total number of NeuN^+^/BrdU^+^ cells in healthy mice or the 6-OHDA mouse model of PD. **g** NeuN (red) BrdU (green), and Hoechst (blue) staining in the striatum of the 6-OHDA mouse model treated with saline or miR-124 NPs. **h** Apomorphine-induced rotation test illustrates net rotation to the contralateral side of healthy or PD mice. Data are expressed as the mean ± SEM (*n* = 4–6 mice) ^#^*P* < 0.05, **P < 0.01, ***P < 0.001. Reproduced with permission from ref. ^132^

Levodopa therapy is also a widely used strategy in AD patients; however, the application of nanoformulations in clinical settings is rare. The chronic administration of levodopa induces dyskinesis, which can be controlled by maintaining the sustained delivery of levodopa. Subcutaneous administration of levodopa-α-lipoic acid (LD-LA)-embedded PLGA-MS in rats has shown the ability to mitigate levodopa-induced motor syndrome side effects. The advantage of a polymeric microsphere matrix lies in its ability to protect the loaded drug from enzymatic and chemical degradation. Interestingly, a single administration of LD-LA microspheres maintained sustained levels of DA in the brain striatum for up to 4 days.^119^ Similarly, a significant reduction in dyskinesis and improvement in PD symptoms and associated biochemical markers (ΔfosB, c-AMP-regulated phosphoprotein or phosphorylated dopamine) were observed in the PD animal model after the administration of levodopa/benserazide encapsulated in PLGA-NPs.^120,121^ The DA receptor agonist rotigotine is employed in the therapy of PD, but its low bioavailability limits its efficacy. Continuous stimulation of the dopaminergic system is an important strategy in the treatment of PD because pulsatile administration promotes motor complications (dyskinesia). Rotigotine-loaded PLGA-MS has been found to be helpful in maintaining an adequate level of rotigotine for an extended duration, which eventually reduces the symptoms of pulsatile levodopa-induced dyskinesis.^122^ Other investigators also confirmed that chronic administration of rotigotine-loaded PLGA-MS in monkey and SD rats showed sustained release behavior accompanied by better safety.^123,124^ In another study, nanocarrier-based transdermal delivery of the DA agonist ropirinole restored deviated oxidative stress markers such as thiobarbituric acid, glutathione antioxidant enzymes and catalase in the PD rat model.^125^ In most of the investigations, the administration of drugs through nanocarriers enhanced the bioavailability, thereby improving the behavioral and biochemical parameters in PD models. SLNs have also shown promise for the sustained delivery of drugs. Sustained delivery and higher bioavailability of ropirinole, bromocriptine and apomorphine could be achieved through SLNs.^126,127^ Furthermore, SLN-mediated bromocriptine and apomorphine delivery in PD rodent models improved akinesia and rotation behavior.^128^ To treat PD, potential growth factors were also delivered through nanocarriers. The regeneration of affected dopaminergic neurons accompanied by functional improvement could be achieved through the implantation of glial cell-derived neurotrophic factor (GDNF)-loaded PLGA-MS in the brains of PD animal models.^129^ Similar results were also observed by Garbayo et al., where PLGA-MS-mediated GDNF release demonstrated antiparkinsonian effects, as confirmed by the improvement in the amphetamine-induced rotational behavior test.^130^ Apart from this GDNF-loaded microsphere increase in TH-positive fibers in the striatum and substantia nigra, the results suggested neuroprotection and neurorestoration activity.^129,130^ Gujral et al. also showed that PLGA/collagen MS may be used to deliver GDNF, and in vitro application suggests that it stimulates the differentiation of neuronal progenitor cells into mature neurons.^131^ Polymeric nanoparticles containing perfluoro-1,5-crown ether mediated miR-124 delivery and increased the number of migrating neuroblasts both in healthy mice and in 6-OHDA mouse models for PD.Neural stem cells of the subventricular zone treated with microRNA miR-124 NPs showed significant proliferation of neuroblasts (Ki67+/DCX+) but a lower number of proliferating glial cells or astrocyte-like cells (Ki67+/GFAP+) than in the control. Thus, the data demonstrate the positive effect of miR-124 NPs, as evidenced by neuroblast proliferation (Fig. 4a, b). Furthermore, decreased expression of SOX-9 and Jagged1 was observed, which promotes neurogenesis by modulating other signaling pathways (Fig. 4c, d). A 2.5-fold increase in the number of mature neurons indicated that miR-124 NPs promote neuronal differentiation. miR-124 NPs also induced the migration of neurons into the compromised striatum of the PD model (Fig. 4f, g). An apomorphine-induced rotation behavior test confirmed the role of miR-124 NPs in ameliorating motor symptoms in a PD mouse model (Fig. 4h).^132^ Briefly, in vitro and in vivo studies suggest a significant role for miR-124 NPs in the treatment of PD (Fig. 4e).

### Nanomedicines in cardiovascular diseases

Cardiovascular diseases (CVDs) are one of the major causes of morbidity and mortality worldwide. Although a long list of therapeutic agents are available in the cardiovascular cohort, their success has been limited by their ability to reach the target tissue. Importantly, nanomaterials avoid rapid renal excretion and remain in circulation for extended durations. This characteristic feature facilitates extravasation through the vascular system and allows nanomaterials to accumulate and distribute well in the desired tissue or organs, thereby achieving maximum therapeutic efficacy at minimum drug dosage.

#### Blood pressure

Blood pressure (BP) or hypertension is the most common disease in the cardiovascular disease cohort. BP is a major risk factor for several serious CVDs, such as stroke, myocardial infarction, and peripheral artery disease.^133^ Although numerous drugs for the treatment of blood pressure are available in the market, they face challenges due to high dosing, low permeability, low bioavailability, and associated side effects.^134^ Since BP follows a circadian pattern, nanomedicines with sustained drug release features may play a major role in controlling BP fluctuations.^135^ The encapsulation of drugs into nanocarriers may overcome these barriers by maintaining the desired drug concentration for an extended duration. Various nanoparticulate systems, such as lipid-based and polymeric nanoparticles, have been found to be effective in overcoming the limitations of conventional (immediate release) anti-hypertensive drugs.^136^ Apart from bioavailability and permeability issues, the pH differences of gastrointestinal tracts (acidic in stomach and basic in the intestine) hinder the pharmacological activity of the drugs. For example, the bioavailability of candesartan cilexetil is affected by acidic pH.^137^ Currently, most commonly used nanomedicines are FDA-approved polymeric nanocarriers conjugated to standard medications for improved absorption and bioavailability. Polymeric nanoparticles, including PLGA, PCL, eudragit, and chitosan, can deliver pH-sensitive drugs.^138^ Polymer-based nisoldipine eudragit S100 nanoparticles released the drug from the polymer at the pH of the colon, which could circumvent drug metabolism in the gut and liver, thereby maintaining the integrity of the drug and extending the availability in systemic circulation.^139^ Furthermore, felodipine (calcium-channel blocker) encapsulated in PLGA NPs showed improved antihypertensive effects because of the increased solubility and bioavailability of the drug.^140^ Similar results were also observed when other commonly used hypertensive drugs (hydrochlorothiazide, amlodipine, and candesartan) were conjugated with PLGA NPs.^141^

Apart from PLGA, other established polymers, such as PLA and chitosan have also been conjugated with antihypertensive drugs for efficient and controlled drug delivery. Several lines of evidence demonstrate the potential of polymeric nanoparticles in regulating BP. PLA magnetic NPs of aliskiren showed better bioavailability and eventually better control of BP.^142^ In another study, Kim et al. reported a significant and sustained reduction in blood pressure using nifedipine encapsulated in PLGA, PCL, and eudragit nanoparticles.^143^ The major advantage of sustained-release anti-hypertensive formulations is their ability to manage BP fluctuations by maintaining high and prolonged plasma drug concentrations. Furthermore, sustained release through drug delivery vehicles requires lower drug dosage than conventional drugs. The minimum plasma concentration obtained with the sustained release formulation of indapamide at the dosage of 1.5 mg/d was similar to that obtained with 2.5 mg/d of conventional drug formulation.^144^ Thus, nanoformulations have important implications in terms of dose reduction and thereby better clinical safety and patient compliance. Liposomal drug formulations have been successfully tested in animal models of hypertension. A single intravenous administration of liposomal formulation encapsulated with vasoactive intestinal peptide, a well-known vasodilator and immunomodulator, has been reported to normalize the systemic BP for longer durations than nonencapsulated peptide.^145^ It is worth adding that administration of this liposomal formulation via other routes (subcutaneous and intratracheal) is also effective in delivering antihypertensive effects.^146^ Similarly, liposomal encapsulation of lercanidipine resulted in significantly improved drug absorption and bioavailability and eventually marked reduction in BP.^147^ Cyclodextrin NPs are also considered potent carriers of drugs, and the hydrophobic cavity of cyclodextrins protects the drug from faster degradation and thereby prolongs the bioavailability. Mariangela et al. demonstrated that the encapsulation of captopril in cyclodextrin nanoparticles had a significant beneficial effect on BP, particularly at lower dosages.^148^ Recently, cyclodextrin complexes were reported to facilitate the beneficial effects of hydrochlorothiazide in a rat model by protecting the drug from rapid hydrolysis.^149^ Nanoparticle-mediated gene silencing is another promising approach to regulate BP, which primarily acts through the siRNA-mediated regulation of gene expression. As such, siRNA is highly prone to degradation and requires a delivery vehicle to circumvent degradation by endo- and exonucleases present in the blood circulation and cells.^150^ Briefly, the successful entrapment of anti-hypertensive drugs by the nanoparticulate system protects the drug in circulation and prolongs the systemic availability of drugs at the desired concentration, which eventually regulates the BP.

#### Atherosclerosis

Atherosclerosis is a well-known clinical condition in which plaque builds up inside the arteries. Furthermore, the hardening and narrowing of arteries due to excessive plaques restrict adequate blood flow and eventually lead to stroke, coronary artery disease, and peripheral vascular diseases. Several features of atherogenesis, such as enhanced vascular permeability, the expression of adhering molecules in endothelial cells, the accumulation of inflammatory monocytes (e.g., Ly6Chigh CCR2+ in mice, CD14highCD16–in humans) or macrophages, and the expression of proteases are utilized for the imaging and therapy of atherosclerosis. The nanotechnology-based imaging of plaque macrophages is utilized to identify high-risk plaques. An iodinated aroyloxy ester, N1177, has been successfully tested in an atherosclerotic animal model for the detection of macrophage accumulation in arterial walls.^151^ Approximately 100-fold higher resolution than conventional computed tomography scans showed promise, and this approach is being clinically tested for application in human subjects.^152^ Furthermore, superparamagnetic iron oxide nanoparticles (SPIO) facilitate the internalization of nanoparticles in macrophages, as evidenced by the incorporation of monocrystalline iron oxide particles in atherosclerotic macrophages.^153^

**Fig. 5:**
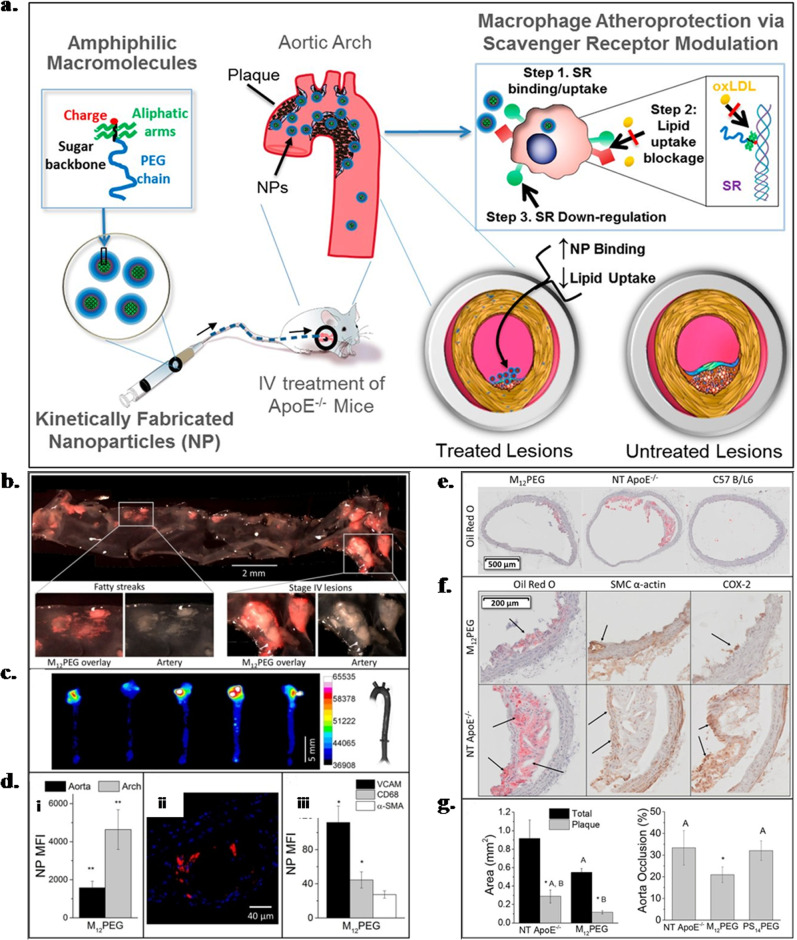
Sugar-based AM NPs (M_12_PEG) showed atherosclerotic lesion-specific localization. **a** Schematic diagram demonstrating the favorable effect of sugar-based AM NPs on atherosclerotic plaque removal. **b** Fluorescence images revealing specific lesion targeting by M_12_PEG (red) at 24 h postinjection. **c** Ex vivo aortic arch fluorescence images demonstrate the highest levels of NP accumulation up to 60 days. **d** NP accumulation (red) within atherosclerotic plaques. Intravenous administration of AM NPs in ApoE−/− mice demonstrated in vivo efficacy by mitigating atherosclerotic endpoints. **e** Reduction in lipid burden and plaque development confirmed using Oil Red O staining. **f** M_12_PEG efficacy at regions of plaque formation in aorta, including the prevention of lipid and cholesterol formation (Oil Red O images), neointimal hyperplasia (smooth muscle cell, α-actin images), and inflammation (COX-2 images), as indicated by the arrows. **g** M_12_PEG resulted in a decrease in aorta occlusion, while the control NP formulation (PS_14_PEG NPs) and ApoE−/− mice showed no improvement. Reproduced from ref. ^154^

Sugar-based amphiphilic macromolecules (AMs) fabricated into serum-stable NPs (M_12_PEG) downregulated the scavenger receptors MSR1 and CD36, resulting in minimal lipid accumulation in an atherosclerosis-prone apolipoprotein E-deficient (ApoE−/−) cardiovascular disease mouse model.^154^ Furthermore, atherosclerotic lesion-specific delivery of NPs was reported in the aortas of ApoE−/− mice. The largest plaque burden in this mouse model is observed in the aortic arch and carotid branch points (Fig. 5a). NPs were found to accumulate at the highest concentration in these aortic areas, as confirmed by fluorescence imaging and corresponding quantitative data (Fig. 5b, c). Confocal microscopy suggests the localization of NPs around the necrotic core of plaques (Fig. 5d). Furthermore, NPs were reported to be highly associated with cells expressing vascular cell adhesion molecules. Aorta cross-sections indicate the presence of plaques with necrotic cores in untreated (ApoE−/−) compared to treated (M_12_PEG) mice. Morphologically, artery occlusion was found to be significantly reduced after treatment. M_12_PEG was also able to mitigate lipid accumulation (Oil Red O), inflammation (COX-2) and neointimal hyperplasia (smooth muscle cell, α-actin), demonstrating its efficacy in atherosclerotic disease (Fig. 5e–g).^154^

SPIO is a type of contrast enhancer that has been utilized in MRI due to its adequate particle size, biocompatible nature, and targeting abilities.^155^ Among polymeric nanoparticles, PLGA and poly-lactic acid (PLA) are promising FDA-approved drug delivery carriers and are currently being used in clinical settings.^156^ Early diagnosis of vulnerable plaques in asymptomatic patients is a primary goal in cardiovascular imaging. Such imaging-based diagnosis could be achieved by FTIC-loaded PLGA nanoparticles, which could be delivered to atherosclerotic sites, most likely via enhanced permeability in the atherosclerotic lesions and mechanistically through phagocytosis by monocytes and macrophages.^157^ Furthermore, pitavastatin (HMG-CoA reductase inhibitor) embedded in PLGA-NPs reduced plaque destabilization/rupture by reducing the circulation of Ly6Chigh monocytes and the infiltration of macrophages into atherosclerotic lesions by interfering with MCP-1/CCR2 signaling-mediated monocyte recruitment.^157^ Liposome-dependent delivery of siRNA against the chemokine receptor CCR2 also inhibited monocyte/macrophage recruitment to the atherosclerotic lesion and arteries.^158^ After systemic administration in mice, CCR2-silencing siRNA accumulated in the spleen and bone marrow and localized to monocytes. Substantial degradation of CCR2mRNA in monocytes inhibited their accumulation at the inflammation site. Moreover, treatment reduced their number in atherosclerotic plaques and reduced the infarct size after coronary artery occlusion.^158^

#### Myocardial ischemia-reperfusion injury

Myocardial ischemia-reperfusion (IR) injury leads to necrosis and apoptosis of cardiomyocytes, particularly through high levels of reactive oxygen species and mitochondrial disturbances. The major reason for the failure of most clinical trials in myocardial IR injury is insufficient drug delivery within a limited therapeutic time window. In this regard, PEGylated liposome-dependent adenosine delivery shows effective cardioprotection by delivering a more adequate adenosine concentration in the ischemic myocardium rat model than does free adenosine.^159^ In cases such as myocardial IR, nano drug delivery vehicles accumulate in the injured tissue due to inflammation-mediated increased vascular permeability.^160^ PLGA nanoparticles have shown promise for use in myocardial IR injury due to their ability to target inflammatory cells and the ischemic myocardium. In a myocardium IR mouse model, administered indocyanine green-loaded PLGA nanoparticles accumulated exclusively in the ischemic myocardium.^161^

**Fig. 6:**
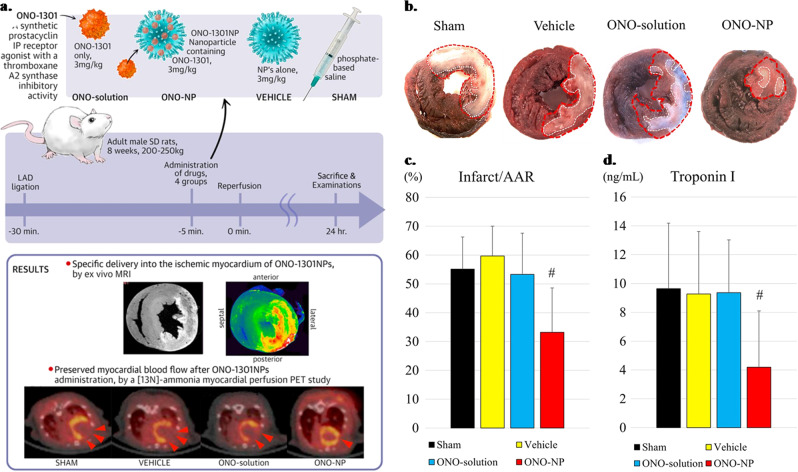
**a** Evans blue and triphenyltetrazolium chloride staining of the heart across the treatment groups (Sham, Vehicle, ONO-solution, and ONO-NP). **b**, **c** The size of the area at risk of infarct (Infarct/AAR) was reduced significantly in the ONO-NP group compared to the other 3 groups. **d** The troponin I level in the plasma was lowest across the group. ^#^Significantly smaller than in the sham, vehicle, and ONO-solution groups. AAR = area at risk. Reproduced from ref. ^162^

Among most recent formulations, intravenously injected ONO-1301 (Ono Pharmaceuticals, Osaka, Japan) containing nanoparticles (ONO-1301NPs) showed selective accumulation and prolonged retention in ischemic myocardial tissue compared to other groups (Sham, Vehicle and ONO-solution) (Fig. 6a).^162^ Myocardial blood flow was significantly improved, accompanied by a significant decrease in infarct area (Fig. 6b, c). Furthermore, upregulated pro-angiogenic cytokines (e.g., vascular endothelial growth factor and angiopoietin-1) were reported in the ischemic myocardium, which preserves the dense vascular network and facilitates myocardial blood flow. ONO-1301 NPs helped downregulate troponin I (Fig. 6d) along with the inflammatory cytokines IL-1β, IL-6 and tumor necrosis factor-α, which eventually led to enhanced myocardial blood flow and reduced infarct size.^162^

### Nanomedicines in respiratory diseases

Nanomedicines involve the use of drug carrier systems for the encapsulation or conjugation of drug/therapeutic compounds. Because of the nanosized structure, nanomedicines are able to reach distant and specific sites of the body.^7^ For respiratory diseases, drugs are introduced to the lung either through inhalation or by systemic routes. It is worth adding that inhalation is an advantageous approach to deliver nanomedicines to the lung because this delivery method minimizes drug resistance, therapeutic dose, and side effects. Polymeric materials have shown tremendous potential as drug delivery vehicles due to their high biocompatibility and biodegradability.^163^ Polymeric nanomaterials entrap the drug efficiently and either allow drug release in a sustained manner or facilitate the release kinetics in response to cellular or extracellular stimuli, such as temperature, pH, and redox potential.^164^ Functionalization of these particles allows the chemical conjugation of various ligands, DNA, peptides, and carbohydrates for the efficient targeted delivery of the particles.^165^

#### Asthma

**Fig. 7:**
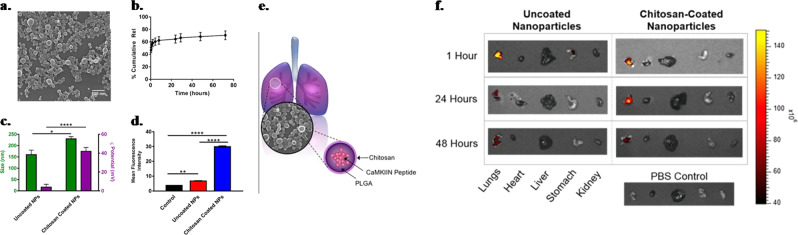
Chitosan-coated PLGA NPs show increased size, zeta potential, and cellular uptake by human airway epithelial cells. **a** SEM image of chitosan-coated PLGA NPs. **b** Percentage cumulative CaMKIIN release from PLGA NPs. **c** Determination of size and zeta potential in chitosan-coated and uncoated PLGA NPs. **d** Cellular uptake of CaMKIIN in HAECs cells using control (cells alone in culture media), uncoated and chitosan-coated PLGA NPs (*P ≤ 0.05, **P < 0.01, ****P < 0.0001). **e** Schematic presentation of chitosan-coated PLGA NPs in lung. **f** Images of control (saline) and uncoated and coated PLGA NPs loaded with fluorescent dye in various organs (lungs, heart, liver, stomach, kidney) at 1, 24, and 48 h after oral administration. Reproduced from ref. ^175^

Asthma is a common chronic disease that is characterized by inflamed lung airways, coughing, wheezing, and shortness of breath. Corticosteroids and bronchodilators are the most widely used therapies for asthma and reduce disease symptoms at the cost of long-lasting side effects.^166^ For example, the long-term use of corticosteroids may result in immunosuppression, predisposing the patients to chronic lung infections.^167^ Thus, alternative therapeutic approaches are needed that could treat the disease without immunosuppressive effects. The use of NPs is a new paradigm for the treatment of respiratory diseases.^168^ NPs facilitate drug delivery directly to the target tissues, thus improving the lung deposition and eventually the therapeutic effects of anti-asthmatic drugs while attenuating the adverse side effects. A wide range of nanoparticulate systems have been evaluated for application in lung diseases, including asthma.^168,169^ These nanomaterials escape from several physiological barriers, such as airway mucus, to cross the endothelium and deliver desired pharmacological effects.^170^ Steroids encapsulated in NPs produce more sustained therapeutic effects at the site of airway inflammation than do free steroids. Salbutamol embedded in NPs was found to deliver sustained relief due to the higher and sustained availability of drugs to the lung membrane. In another study, the liposome-mediated delivery of salbutamol sulfate prolonged the therapeutic effects up to 10 h by increasing the concentration and retention time in the lungs.^171^ Adjunct therapy of curcumin as a formulation of SLNs is able to enhance the bioavailability and therapeutic efficacy of this anti-inflammatory compound in asthma patients.^172^ Montelukast, a leukotriene receptor antagonist, is used to prevent wheezing, bronchospasm, and asthma-induced coughing. Inhalable montelukast-loaded nanostructured lipid carriers have been found to improve systemic bioavailability (12-fold) and eventual therapeutic outcomes compared to those of montelukast aqueous solutions. It is worth noting that the inhalation route offers several advantages over the parenteral or oral route. Inhalable drugs reduce dosing frequency, and bypassing the first pass hepatic metabolism reduces hepatocellular toxicity.^173,174^ Reports suggest the overexpression of Ca^2+^/calmodulin-dependent protein kinase II (CaMKII) in asthmatic patients. CaMKII inhibition reduced disease phenotypes of allergic asthma, providing the rationale for targeting CaMKII as a potential therapeutic approach for asthma. CaMKII inhibitor peptide (CaMKIIN)-loaded NPs mitigated the severity of allergic asthma in a mouse model. Nanosized chitosan-coated PLGA NPs were reported to have the ability to sustain the release of CaMKIIN with effective cellular uptake (Fig. 7a–c). Further high cellular uptake was observed with the use of nanosized chitosan-coated PLGA NPs, as confirmed by the fluorescence intensity results (Fig. 7d). These chitosan-coated NPs specifically localize in the lungs and fluoresce up to 48 h, which suggests that NPs are able to release CaMKIIN with higher bioavailability and high retention time (Fig. 7e, f).^175^

Telodendrimer is a class of nanocarrier that has been reported to have higher loading capacity, higher stability and slower release of drug formulations than other types.^176^ The telodendrimer-mediated sustained release of dexamethasone has been found to be effective in decreasing allergic lung inflammation compared to equivalent doses of dexamethasone alone.^177^ A wide range of lipid- and polymer-based vectors has been employed to deliver nucleic acid as a therapeutic agent in pulmonary diseases.^178^ The application of chitosan^179^ and PLGA^180^ has also been employed in the pulmonary delivery of nucleic acids. The use of chitosan interferon-γ-pDNA NPs reduced airway hyperresponsiveness in an allergic asthma mouse model.^181^ In another study with a similar mouse model, pDNA NPs conjugated with cysteine-linked poly-L-lysine and poly(ethylene glycol) were found to be efficient in the delivery of thymulin, a well-known nonapetide with anti-inflammatory and antifibrotic effects. A single instillation of pDNA NP-mediated thymulin improved lung function for up to 27 days by preventing lung inflammation, muscle hypertrophy, and collagen deposition.^182^ In recent times, biodegradable DNA NPs have shown potential for inhaled lung gene therapy, which is capable of overcoming the mucus barrier of lung airways.^183^

#### Chronic obstructive pulmonary disease

Chronic obstructive pulmonary disease (COPD) is characterized by an abnormal inflammatory response to inhaled noxious particles or gases, leading to chronic inflammation of the airways and airflow limitation accompanied by destruction of the lung parenchyma. The current treatment strategy for COPD includes inhaled corticosteroids, anticholinergics, and β2-agonists, which have been primarily effective in controlling the symptoms but do not cure the underlying disease.^184^ The major challenges in the nanotherapeutics of COPD are airway defense, mucus hypersecretion and severe inflammation. In this scenario, a potential multifunctional polymeric vesicle composed of PLGA and PEG has been suggested for the delivery of COPD drugs such as prednisolone (corticosteroid) and theophylline (bronchiodilator).^185^ Furthermore, dimethyl fumarate, an antioxidant Nrf2 activator, is capable of reaching the lower airways to treat inflammation in this region.^186^ Based on several investigations, metallic NPs such as Au and TiO_2_ have been suggested as potential nanocarriers for COPD treatment.^187,188^ Noninvasive magnetic resonance imaging of macrophage subpopulations is possible using antibody-conjugated SPIO NPs. Anti-CD86 and anti-CD206 antibody-conjugated SPIO NPs were instilled in COPD mouse models via the intrapulmonary route, and the NPs demonstrated specific adherence to the proinflammatory (M1) and resolutive (M2) macrophage subpopulations. Such noninvasive nanoparticle-mediated high-contrast imaging methods may be promising for the diagnosis of pulmonary inflammation.^189^ A formulation of PEGylated immunoconjugated PLGA-nanoparticles demonstrated the targeted delivery of ibuprofen (an anti-inflammatory drug) to neutrophils, which inhibited neutrophilic inflammation in a lipopolysaccharide-induced murine model of obstructive lung disease.^190^

#### Pulmonary tuberculosis

Tuberculosis (TB) is caused by *Mycobacterium tuberculosis* (MTB) and is one of the leading causes of death worldwide. Although the occurrence of this infectious disease is global, the highest prevalence is reported in Asia, Africa, and South America.^191^ In fact, MTB affects all organs of the human body, but high incidences are reported in the lung because the primary route of infection is the inhalation of MTB from close contact with infected human subjects.^192,193^ MTB can reach the lung alveoli, where the cells are phagocytized by alveolar macrophages (AMs). MTB resists macrophage-mediated bactericidal mechanisms by preventing phagolysosome formation.^194^ Therefore, MTB can multiply and spread to other organs of the body, resulting in extrapulmonary TB.^195^ According to an estimate, one-third of the human population is latently infected with MTB. However, only 5–10% of latent infection cases may progress to an active form of disease. Some conditions, such as HIV and diabetes, are associated with a high risk of susceptibility to MTB infection. Apart from conventional oral drug delivery, nano drug delivery systems will provide an opportunity to exploit the nasal delivery of anti-TB drugs directly to the lungs. This approach has the advantage of achieving pharmacologically effective drug concentrations in the AMs, which ensures better treatment outcomes. Furthermore, the nanodrug delivery system reduces the adverse systemic side effects and frequency of drug administration, which eventually leads to better patient compliance.^196,197^ Currently available chemotherapy includes several lines of treatment depending upon the severity of TB. First-line drugs, such as rifampicin (RIF), isoniazid (INH), pyrazinamide (PZA), and ethambutol, are used either alone or in combination with second-line drugs, such as injectable agents (streptomycin, amikacin, kanamycin, viomycin, and capreomycin), fluoroquinolones (levofloxacin and ofloxacin) and other oral agents (cycloserine, ethionamide, prothionamide, terizidone, and paraamino salicylic acid).^198^ Although several drugs have been discovered for TB treatment, new drugs are always needed to fight drug-resistant TB.^199^ Currently, several new drug candidates are in clinical trials. Delamanid and bedaquiline have shown promise to fight against multidrug resistance (MDR) TB.^200^ Moreover, the current treatments have limited effects on MTB and are accompanied by adverse side effects. In addition, repeated higher doses of drugs are needed to reach therapeutic levels, which is one of the major reasons for MDR-TB.^201^

Although the oral route is the most convenient and least expensive, repeated administration of high doses is required to achieve therapeutic levels of anti-TB drugs due to rapid hepatic first-pass metabolism and reduced gastro-intestinal absorption. Other disadvantages of the oral route are the high systemic exposure and adverse side effects. On the other hand, the parenteral and pulmonary routes have higher bioavailability due to bypassing the first-pass metabolism.^196^ In this context, the inhalation route is advantageous for the pulmonary delivery of anti-TB drugs and requires lower doses to achieve therapeutic effects. The pulmonary delivery of drugs is a convenient and effective approach for the treatment of TB.^202^ In this context, the encapsulation of drugs into nano drug delivery systems offers a number of potential benefits to penetrate and cross the biological barriers to reach the targeted sites in the lungs. Furthermore, the phagocytic nature of AMs is an added advantage for targeted delivery in lungs.^203^ Mesoporous silica nanoparticles (MSNPs) can be adapted as a platform for the delivery of anti-TB drugs. Drug-specific surface functionalization has been employed for RIF, and further functionalization with poly(ethylene imine) (PEI) yields high loading and controlled drug delivery to MTB-infected macrophages compared to uncoated MSNP.^204^ On the other hand, in the case of INH, MSNP decorated with pH-operated nanovalves has been designed to open after endocytosis and endosome acidification to deliver the drug directly to MTB-infected macrophages. Thus, functionalized MSNP may act as a controlled drug delivery vehicle for tuberculosis treatment. Functionalized MSNP can be internalized efficiently, as confirmed by using human macrophages such as THP-1 cells. The majority of the macrophages that were incubated with RITC-labeled NP-RIF internalized the fluorescent nanoparticles and exhibited 10-fold higher fluorescence intensity than the untreated control macrophages. Approximately 90% of MSNP was intracellularly colocalized with CD63 after 3 h of incubation.^204^

The physicochemical properties of drugs encapsulated in nanoparticles are the most important characteristic for achieving a proper drug distribution in lungs. A particle size below 1 μm is suitable to deliver the drugs to the pulmonary alveoli.^205^ The use of neutral NPs as drug delivery vehicles is the most common approach in anti-TB therapy.^206^ Ciprofloxacin encapsulated in liposomes has been employed in anti-TB therapy, offering pulmonary delivery through nebulization. Finlay et al. showed high loading efficiency (90%) of liposomes made of phosphatidylcholine (PC) and Chol, of which up to 30% remained encapsulated after nebulization.^207^ In another investigation by the same authors, the best loading efficiency (nearly 100%) was obtained by a negatively charged phospholipid, dimyristoyl phosphatidylglycerol.^207^ Furthermore, Bhavane et al. developed ciprofloxacin encapsulated in liposome agglomerates made of 1,2-dipalmitoyl-sn-glycero-3-phosphatidylcholine (DPPC), cholesterol and mPEG-DSPE or DPPC, cholesterol and distearoylphosphoethanolamine amino (polyethylene glycol) conjugates. Using this formulation, the administration of a single dose was sufficient for an extended period of time.^208^ The progressive release of the drug mitigated the inflammation significantly more effectively than free ciprofloxacin.^208^ DPPC has also been employed for INH delivery; however, the loading efficiency was found to be lower (~37%).^209^ However, the formulation was biocompatible, and sustained release was maintained for up to 24 h with a burst release of 50% in the first 5 h.^209^ In another study, RIF encapsulated in liposomes (made of PC and Chol) as aerosols showed a sustained release profile with an encapsulation efficiency of ~30%.^210^ In a recent investigation, Patil et al. suggested that drug release decreases with increasing concentrations of chol, as confirmed using rifampicin-loaded freeze-dried liposomes.^211^ This formulation was optimized with a drug entrapment efficiency of 79%, and a sustained release pattern could be achieved showing higher anti-TB activity than that of the pure drug.^211^ Targeted delivery of rifampicin could also be achieved by using liposomes coated with macrophage-specific ligands. Macrophage-specific ligands such as MBSA, O-SAP, and DCP facilitate the preferential accumulation of formulations in lung macrophages. This approach reduces systemic and local toxicity and provides better results than uncoated formulations.^212^ Similarly, rifapentine-loaded proliposomal dry powder was effective in the direct delivery of the drug to the lungs and eventually had a better therapeutic effect.^211^ These studies clearly indicate the remarkable potential of liposomes in the direct lung delivery of anti-TB drugs.

Lipid NPs show higher stability and drug loading efficiency with appropriate sizes and shapes for direct lung targeting and controlled drug delivery.^213^ Additionally, it is also possible to change the surface chemistry of lipid NPs to achieve active targeting of alveolar macrophages of the lung. Mannose is a common ligand associated with lipid NPs.^214^ SLNs have been extensively used in formulations for the delivery of anti-TB drugs. Jain et al. used different nanocarriers for ciprofloxacin encapsulation and showed that SLNs were able to promote a prolonged drug release.^215^ Apart from ciprofloxacin, SLNs were also capable of loading other anti-TB drugs, such as rifabutin, INH, RIF, and PZA.^215^ A mannose coating on SLNs made of tristearin has been found to be suitable for drug delivery, and the cellular uptake of rifabutin was enhanced up to six-fold compared to that of uncoated formulations.^216^ Furthermore, in vivo studies confirmed the higher accumulation of rifabutin in lungs with less immunogenicity for mannose-coated formulations.^216^ In a similar approach, Pandey and Kuller demonstrated that SLNs made up of stearic acid may also be used as potential delivery vehicles for INH, RIF, and PZA. Interestingly, all these formulations showed sustained release for up to 72 h with an initial burst drug release pattern of less than 20% in the first 6 h. The pulmonary delivery of drugs encapsulated in SLNs through nebulization resulted in specific deposition in the lungs and the detection of the drug in other organs for up to 7 days.^217^ Jain and Banerjee also demonstrated the sustained release of ciprofloxacin (up to 80 h).^215^ Further RIF-loaded SLNs have been found to be suitable for AM targeted delivery due to the specific internalization of nanosized SLNs into AM.^218^

### Cancer and challenges in its therapy

Cancer, usually termed malignant tumors, encompasses a large number of diseases where mutations in the genetic material of the cell are responsible for uncontrolled growth. The impact of cancer and the dismal prognosis for patients include a high mortality rate, poor quality of life and expensive therapy. Much technological development and much research have been undertaken to fight against cancer, such as chemotherapy, injections of micro/nanoparticles, immunotherapy, and radiation therapy. However, these technologies possess some defects associated with systemic delivery, such as poor/low drug concentration at the tumor site, nonselectivity towards cells or tissues leading to toxicity, and the low efficacy of the drugs due their short half-life. In local delivery systems, the drug is delivered directly at the targeted site via implantation. The main advantage of local therapy is that the pharmaceutical concentration of chemotherapeutics in the tumor environment can be maximized, and the toxicity to other nontargeted organs can be minimized. Furthermore, local delivery can enhance the efficacy of the drug by avoiding the long journey through a hostile environment required to the target site when the drug is delivered systemically.^219^ To better understand the tumor and increase the availability of drugs, several carriers have been explored that can deliver drugs in a controlled manner without causing side effects.^220^ So far, versatile materials are used, including polymers,^221^ lipids,^222^ inorganic molecules, hydrogels,^223^ and macromolecular scaffolds,^224^ which have led to the development of systems that deliver chemotherapeutics directly to the tumor sites with improved therapeutic efficacy.^7^

#### Enhanced permeation effect in tumors

Common features of tumors include leaky vasculature and poor lymphatic drainage. Several methods have been proposed for targeting tumors, e.g., the enhanced permeation and retention effect where the leaky vasculature promotes the permeation of small molecules and poor lymphatic drainage allows the retention and accumulation of chemotherapeutics in the tumor. In contrast to easily diffusible free drugs, a nanocarrier can penetrate into the tumor tissue through the leaky vasculature due to the enhanced permeation and retention effect.^225^ In the enhanced permeation effect (EPR), the delivered agent is released locally and rapidly taken up by cancerous cells to accomplish its function. Targeted tumor cells express or overexpress specific receptors or antigens that can be targeted using ligands such as antibodies or small peptides that recognize and bind to them.^226^ Smaller molecules accumulate in tumors faster than larger molecules, but larger molecules can be retained for longer times within the tumor. Therefore, size is a dominant factor for controlling drug accumulation at the tumor site. In one of the reports, liposomes 90 nm in diameter could escape from the leaky tumor vasculature but did not permeate away from the tumor for a week.^227^

#### Nanocarriers for targeted therapy

Active targeting refers to ligand-receptor interaction after nanoparticles reach the targeted site via systemic circulation. Ligand-receptor interaction is possible only if the two components are in close proximity (< 0.5 nm).^228^ Active targeting in tumors can be achieved by functionalizing the nanoparticles with proteins, peptides, nucleic acid aptamers, carbohydrates, and other small molecules. Several classes of materials have been developed to date for targeted therapy, including biodegradable polymers, liposomes, dendrimers, nanoshells and nucleic acid-based NPs.^2^ In cancer therapy, biodegradable nanoparticles are extensively utilized due to their high biocompatibility.^229^ In targeted delivery, sustained release and site-specific delivery are the prime requirements. Another important factor is the stability of nanoparticles for longer retention in blood circulation and finally accumulation in tumors.^230^ A well-known type of 2D nanocarrier, layered double hydroxides, has drawn attention for its potential as a drug carrier due to its high drug-loading capacity, biocompatibility, and anion exchange capability. The internal and external surfaces of layered double hydroxides (LDHs) can easily be modified for the incorporation of targeting ligands, and their high specific surface areas make them advantageous for diverse applications. Sudipta et al. prepared a series of magnesium and aluminum LDHs through an anion exchange method and incorporated raloxifine hydrochloride, a potent anticancer drug. The drug release was controlled, and higher cytotoxicity was observed in HeLa cells, which was further confirmed through in vivo tumor suppression in a murine model, indicating its application as a drug delivery vehicle.^1,231^ Polymeric nanoparticles can be formulated for the encapsulation of various hydrophilic and hydrophobic smaller molecules, particularly drugs and macromolecules such as proteins or peptides.^232^ The drug release from these nanoparticles can be controlled via diffusion or swelling followed by diffusion or bulk erosion in a time-dependent manner. The rate of release can be governed through polymer modification, the development of new polymers, or the synthesis of copolymers.^233–235^ The main benefits of these biodegradable polymers are maintaining the drug concentration in the optimum range for a longer period of time and thereby increasing the efficacy of the drug and improving patient compliance. Targeted therapy is thus the combination of targeted delivery with sustained drug release, which allows the maximum utilization of the drug delivered at the tumor site.^236^

Liposomes are amphiphilic in nature and are composed of natural or synthetic lipids.^237^ Liposomal formulations with anticancer drugs have been approved by the FDA for human use. Ogawara et al. investigated the effect of PEGylated liposomal doxorubicin (Doxil) in mice with colon cancer or their doxorubicin (DOX)-resistant subclone, which overexpresses P-gp efflux pumps. The results showed potent antitumor effects on DOX-resistant and non-DOX-resistant C26 cells.^238^ In one of the reports, PEGylated hybrid polymer-lipid liposomes were loaded with curcumin and paclitaxel to examine the therapeutic effect of these drugs in combination. For this purpose, paclitaxel-loaded albumin nanoparticles were encapsulated in PEGylated hybrid liposomes containing curcumin by a thin film hydration approach. The sustained release of paclitaxel and curcumin occurred in sequential kinetics, and curcumin downregulated nuclear factor NF-kB and enhanced the therapeutic efficacy of paclitaxel.^239^ Liposomes conjugated with paclitaxel and A7R peptide inhibited tumor growth and angiogenesis simultaneously.^240^ DaunoXome (daunorubicin liposomes) showed better therapeutic efficacy against Kaposi’s sarcoma and other tumors.^241^ Dendrimers are one of the important classes of drug carriers and operate by encapsulating drugs in their unique macromolecular architecture.^242^ Novel water-soluble and biocompatible dendritic systems have been developed using 2,2-bis(hydroxymethyl)-propanoic acid as a drug carrier for doxorubicin.^243^ Zhong et al.^244^ have prepared DOX conjugated dendrimers using polyamidoamine of generation 4 and DOX through acid-sensitive hydrazone bonds. These DOX conjugated dendrimers, when administered to mice bearing melanoma (B16-F10), reduced the tumor burden through enhanced accumulation and tumor penetration. Li and coworkers showed the size effect of pH-sensitive nanoparticles composed of dendrimer building blocks with better tumor penetration and their dissociation into smaller sizes in the tumor environment.^245^

Micelles are also a promising delivery vehicle in cancer chemotherapy. The encapsulation of drugs in micelles is achieved through various physical, chemical or electrostatic interactions.^246^ To enhance internalization, the hydrophilic shell of micelles is grafted with various targeting ligands that recognize the overexpressed receptors in cancer cells. A multifunctional star-shaped micelle has been prepared through the self-assembly of a four-arm poly(ε-capro-lactone)-poly(ethylene glycol) copolymer, where the hydrophilic and hydrophobic segments are connected with disulfide bonds. The coupling of hydrophilic terminal groups with folate units for active targeting showed high stability and exhibited sustained release.^247^ Preeti et al. reported cholesterol-conjugated PLA-based polymeric micelles (mPEG-PLA-Ch) for the efficient encapsulation and delivery of curcumin at the tumor site. The conjugation of cholesterol altered the particle size and enhanced the encapsulation efficiency. Curcumin-embedded mPEG-PLA-Ch micelles significantly reduced the tumor volume in the B16-F10 xenograft tumor model compared to that obtained with pure curcumin.^248^ Combinatorial therapy of paclitaxel (PTX) and alkylated cisplatin prodrug loaded in amphiphilic block copolymer poly(2-methyl-2-oxazoline-block-2-butyl-2-oxazoline-block-2-methyl-2-oxazoline) (P(MeOx-b-BuOx-b-MeOx) in ovarian and breast cancer showed sustained drug release and resulted in improved pharmacokinetics and improved dual drug delivery at the tumor site.^249^

The application of hydrogels has been extensively explored for controlled drug release. In situ gelation of hydrogels is the prime factor in their success as a drug delivery vehicle.^250^ Polyurethane-grafted chitosan-based brush hydrogels have been used as injectable hydrogels in tissue engineering.^251^ In another approach, chitosan nanohybrid scaffolds with enhanced mechanical properties have been reported as drug carriers for biomedical applications. Highly porous, 3D-interconnected nanohybrid scaffolds/hydrogels controlled drug release and were biocompatible as well. The scaffold has been found to be safe and very effective in bone regeneration compared to pure chitosan.^252^

Microgels loaded with DOX showed anticancer activity against HeLa cells, and supramolecular hydrogels of α CD- and polyethylene-modified gold nanocrystals exhibited sustained drug release via host-guest interaction.^253^ Chen et al. used HMDI in a Pluronic F127 polymer for their interconnection. Thermogelation occurred at 37 °C and was stable for 30 days, showing sustained release for a longer period of time. This thermosensitive HDI-PF127/HA hydrogel possessed adequate physiochemical and biological properties and was used as a delivery carrier for the sustained release of anticancer drugs in cancer therapy.^254^

**Fig. 8:**
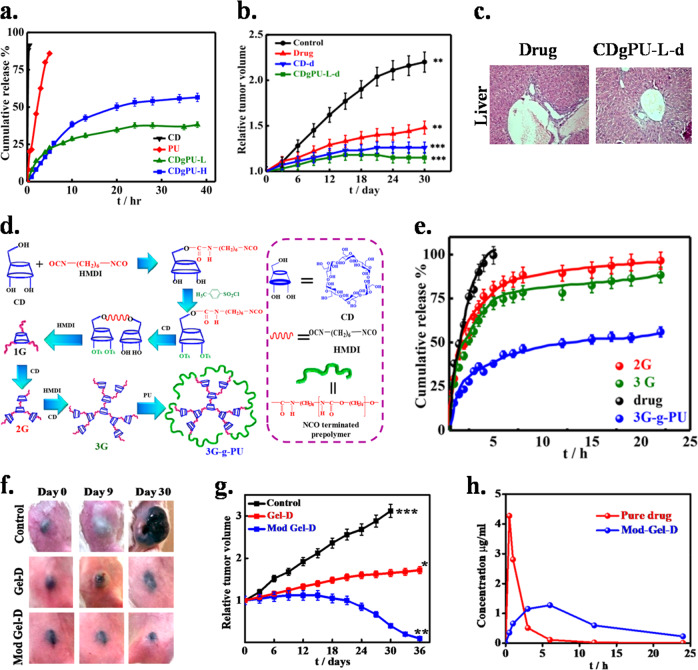
Different polymeric architectures for controlled release and tumor treatment. **a** In vitro drug release from different generations of CD and its grafting to polyurethane. **b** Relative changes in the tumor volume with time treated with indicated patch systems. **c** Histopathological images of hematoxylin and eosin-stained liver in treatment groups. **d** Polymer design and preparation of different generations from CD and linear polymers. **e** Cumulative release percentage of paclitaxel from CD, subsequent generations presenting a sustained release of the drug from a superstructure. **f** Images of mice tumor at the initial day and after treatment with Mod-Gel-d and Gel-d at different time intervals. **g** Relative changes in the tumor volume for different gels with time. **h** Plasma paclitaxel concentration versus time for Mod gel-d and pure drug after intravenous administration. Reproduced from ref. ^255,256^

In conventional chemotherapy, it is difficult to achieve therapeutic concentrations at the tumor site through systemic delivery. Therefore, a carrier incorporating the drug, administered at the tumor site either through a patch or subcutaneously, as shown in Fig. 8, could locally deliver the drug and maintain an appropriate therapeutic concentration in a controlled fashion. Designing different polymeric architectures for controlled release and melanoma treatment has been reported by Shukla et al., where CD-based polymers of different graft densities have been prepared to control drug release and better tumor treatment. Sustained release was observed from the developed copolymers against fast release from the pure drug (Fig. 8a).^255^ Drug-loaded systems efficiently killed the cancerous cells of tumor, revealing the real efficacy of sustained release (Fig. 8b). Treatment of mice bearing melanoma with developed patches of drug-loaded graft copolymers showed significant tumor suppression compared to the effect of the pure drug, where slight suppression was noticed arising from its burst release. Minimal side effects upon treatment were revealed from histopathological images of vital organ (Fig. 8c). A CD superstructure designed through urethane linkages connected the CD to form the hydrophilic core and wrapped the core with polyurethane through grafting (Fig. 8d). Sustained release of paclitaxel from this superstructure was observed, as shown in Fig. 8e, which ultimately resulted in extensive cancer cell killing compared to low killing from the fast release of the pure drug. Injectable gel of this superstructure, when placed subcutaneously in melanoma mice, showed complete shrinkage, which has a greater effect than that of the pure drug, and none of the side effects observed in conventional chemotherapy occurred. Treatment of mice bearing melanoma with developed systems of drug-loaded injectable gels showed significant tumor suppression compared to the effect of the pure drug, where slight suppression was noticed arising from its burst release (Fig. 8f, g). The biodistribution study of the pure drug and gel showed the efficacy of sustained release, since the drug is retained for a longer time period from Mod-Gel-d while it is rapidly eliminated in the pure form as a drug (Fig. 8h).^256^ Therefore, complete melanoma shrinkage with higher biodistribution of the drug through a gel placed subcutaneously is an effective carrier for cancer treatment. However, drugs embedded in nanoparticles, whether inorganic or organic, exhibit sustained drug release and thereby exhibit much greater therapeutic effects than the pure drug.

## Conclusion and future perspective

The application of nanomedicines has witnessed an unprecedented increase during the last decade. The rationale for developing nanotechnology-based drugs includes factors, such as the need for improved versions of current drugs, site-specific or targeted drug delivery and eventually better patient compliance. Nanotechnology-based diagnosis and treatment strategies have been successfully delivered in both preclinical and clinical trials. The advantage of nanomedicines over conventional treatment includes minimum toxicity and maximum efficacy as a result of controlled drug release and improved pharmacokinetics and pharmacodynamics. A suitable drug carrier is important to ensure site-specific sustained drug delivery. Although most of the preclinical studies have been reported to be successful, clinical translation is a major challenge that requires a large number of well-defined clinical trials in various disease cohorts. Nevertheless, several nanomedicines have been developed and marketed for human use, such as Abraxane® (paclitaxel), an albumin-bound paclitaxel formulation for the treatment of cancer; liposome-based drugs such as Caelyx®, Myocet® (doxorubicin), and Mepact® (mifamurtide); and nanoparticle-based therapeutic agents, such as Emend® (aprepitant) for nausea and vomiting and Rapamune® (sirolimus) for graft rejection. In contrast to conventional medicines, a different regulatory framework is needed for nanosystems/nanomedicines because current regulations are no longer appropriate to ensure the safety and quality of emerging nanomedicines. Furthermore, the rapid development of appropriate delivery systems is inevitable to meet current medical needs and to improve on previous nanosystems. Apart from drug delivery, a new generation of nanosystems is focusing more on the delivery of peptides, nucleic acids and genes; however, an extensive benefit-risk assessment is highly desirable before approval for human use.

The one-size-fits-all approach is the major drawback in the clinical translation of nanomedicines. Although an extensive experimental protocol is followed at the preclinical stage, standardization of dosage and a more extensive toxicology profile assessment of nanomedicines are also essential in clinical trials. Since nanoencapsulation increases the circulation half-lives and drug retention in the body, short-term and long-term side effects must be assessed before its authorized application in human diseases. A strong positive benefit to risk ratio is essential for the clinical translation of nanomedicines. The side effects must be assessed particularly in the liver, kidney, spleen, lungs, bone marrow, and lymph nodes, which are major organs where nanomedicines accumulate following systemic administration. Reports also suggest that the cytotoxicity characteristics of nanomedicines may induce macrophage destruction with the possibility of immune suppression. Thus, addressing these aspects is necessary to ensure the safety and utility of nanomedicines in clinics. Additionally, further research is required for the development of synthesis protocols, as for large-scale production, preparation methods should be simple, cost effective and have high batch-to-batch reproducibility. Considering nanotechnology as the most promising area for diagnosis and therapy, additional clinical trials must come from all over the world.
